# Determination of quality of life in adolescents with idiopathic scoliosis subjected to conservative treatment

**DOI:** 10.1186/1748-7161-5-21

**Published:** 2010-09-28

**Authors:** Angelo G Aulisa, Vincenzo Guzzanti, Carlo Perisano, Emanuele Marzetti, Alessandro Specchia, Marco Galli, Marco Giordano, Lorenzo Aulisa

**Affiliations:** 1 Department of Orthopaedics, Children's Hospital Bambino Gesù, Institute of Scientific Research, P.zza S. Onofrio 4 - 00165 Rome, Italy; 2 Department of Orthopaedics, Catholic University of the Sacred Heart, University Hospital "Agostino Gemelli", L.go F. Vito, 1 - 00168 Rome, Italy; 3 Department of Aging and Geriatric Research, Institute on Aging, Division of Biology of Aging, University of Florida, Gainesville, FL 32610-0143, USA; 4 Department of Geriatrics, Gerontology and Physical Therapy, Catholic University of the Sacred Heart, University Hospital "Agostino Gemelli", L.go F. Vito, 1 - 00168 Rome, Italy; 5 University of Cassino, Strada Folcare, 4 - 03043 Cassino (FR), Italy

## Abstract

**Introduction and objectives:**

Physical deformities caused by adolescent idiopathic scoliosis (AIS) coupled with conservative treatment of AIS with orthesis unavoidably impacts on patients' quality of life (QoL). The present study aimed at evaluating the QoL in patients affected by AIS treated with brace. The study also sought to determine the ability of different QoL questionnaires to monitor QoL over the course of treatment.

**Materials and methods:**

Data were collected in 108 consecutive patients (96 females, 16 males) affected by AIS admitted to the outpatient orthopaedic clinic of the Catholic University of the Sacred Heart in Rome (Italy). Patients were subjected to full-time (i.e., 22 hrs per day) conservative treatment with the progressive action short brace (PASB), the Lyon brace or a combination of PASB + Lyon brace. Three instruments were used for QoL determination: the Scoliosis Research Society 22 (SRS-22), Bad Sobernheim Stress Questionnaire (BSSQ) and the Brace Questionnaire (BrQ).

**Results:**

A significant correlation was detected among the 3 scores (p < 0.001). The BrQ possesses a higher capacity to detect changes in QoL in relation to the patient gender, type of brace, curve severity at baseline and at the completion of treatment, and curve type. Overall, boys displayed a higher QoL than girls. In all 3 questionnaires, higher QoL scores were determined in patients treated with the PASB compared with those using the Lyon brace. QoL scores were significantly correlated with the curve severity. Higher QoL scores were obtained by participants with thoraco-lumbar curves as compared with those with other curves.

**Conclusions:**

The 3 questionnaires are effective in capturing changes in QoL in AIS patients subjected to conservative treatment. However, the BrQ possesses a higher discriminatory capacity compared with the other questionnaires tested. PASB-based treatment is associated with better QoL than the Lyon bracing.

## Introduction

According to the WHO, "quality of life is defined as an individual's perception of their position in life in the context of the culture and value systems in which they live and in relation to their goals, expectations, standards and concerns" [1]. Hence, quality of life (QoL) encompasses several dimensions, including the health status and the individual's ability to function in physical, psychological and social domains. In addition, QoL is influenced by the person's ability to enjoy life and achieve expectations and personal development weighted by their importance to the individual. Health-related QoL (HRQoL) is a subset of QoL encompassing domains that are closely related to health and healthcare. This parameter is becoming increasingly important in health policy and clinical decisions.

Adolescent idiopathic scoliosis (AIS) is a complex and progressive condition, significantly affecting patients' QoL. At the time of diagnosis, patients are typically in fairly good health and are usually not aware of the natural history of untreated scoliosis. The primary aims of treatment in AIS are to halt the progression of deformities and improve aesthetic appearance and QoL [2]. Over the last few decades, early diagnosis of AIS, improvements in orthotic treatment and advances in surgical techniques have markedly reduced the prevalence of severe deformities. However, brace-based treatment significantly interferes with several aspects of patients' life [3], which may determine high levels of stress and negatively impact on every-day life [4-6]. In fact, AIS patients, especially those subjected to conservative treatment, may experience social isolation, depression and reduced participation in leisure activities [7]. As a result, the prevalence of psychological disorders may be as high as 19% [8]. Therefore, scoliosis is currently recognized as an important risk factor for psychological discomfort and poor QoL, especially in brace-treated patients. As such, psychological support should always be provided to AIS patients, through group therapy as well as individual counseling, in order to promote disease and treatment acceptance and minimize psychological discomfort [9]. In this scenario, evaluation of QoL should be considered as an important part of AIS treatment and should take into account several factors, including age, gender, familial background, disease acceptance, curve severity, type of bracing, daily duration of treatment, aesthetic abnormalities, and changes in lifestyle.

The SF-36 has been the first instrument employed for QoL evaluation in AIS patients. However, this questionnaire is not disease-specific and might therefore be inadequate to capture scoliosis-related changes in QoL. For this reason, other questionnaires have been proposed, specifically targeted to scoliotic populations, including the Scoliosis Research Society 22 (SRS-22) [10-13], the Brace Questionnaire (BrQ) [4] and the Bad Sobernheim Stress Questionnaire (BSSQ) [5,6].

In the present study, we sought to determine QoL in AIS patients treated with bracing, through the administration of the 3 questionnaires listed above (SRS-22, BSSQ and BrQ). Results were evaluated in relation to demographic as well as scoliosis-related parameters, including age, gender, type of brace, curve severity and treatment effectiveness, in order to determine which tool was the most effective in monitoring QoL in AIS patients.

## Materials and methods

### Study population

A prospective study was conducted in 108 consecutive AIS patients (92 females, 16 males) admitted to the outpatient clinic of the Department of Orthopaedics and Traumatology at the University Hospital "Agostino Gemelli", Rome (Catholic University of the Sacred Heart, Italy). Informed consent was obtained for each participant. Subjects with non-idiopathic scoliosis or severe systemic diseases and those not wearing the brace full-time were excluded from the study.

Participants presented with single thoracic curves (n = 31), single lumbar curves (n = 12), single thoraco-lumbar curves (n = 48), or thoracic and lumbar curves (n = 17). The mean age at the time of questionnaire administration was 15.4 ± 0.2 years (range: 9-18 years). The Risser score at the beginning of treatment was 0-2. The mean curve amplitude at baseline was 32.1 ± 1.0° Cobb (range: 18-70°; median: 30.0°), including 11 patients with surgical curves (46-70° Cobb) who had previously refused surgical treatment, and 18.2 ± 1.1° Cobb (range: 0-55°; median: 15.0°) at the time of interview. The difference in Cobb degrees between baseline and the time of questionnaire administration was -13.9 ± 0.7° (range: -30/-15°; median: 14.0°).

### Types of treatment and curve assessments over time

Participants were treated with Progressive Action Short Brace (PASB, n = 39), Lyon brace (n = 58) or a combination of PASB and Lyon brace (n = 11). The combined approach was prescribed in cases of stiff thoraco-lumbar curves to optimize the hump remodeling. These patients were required to wear the Lyon brace when at home and the PASB while outdoors. Full-time (i.e., 22 hrs per day) bracing was prescribed in all cases. Treatment compliance was determined via patient as well as family interviews. In order to maximize the compliance, each participant was always followed by the same physician. Furthermore, checks were performed every 2 months until a Risser score of 3 was achieved, and every 3 months thereafter.

Curve progression was defined as an increase ≥ 5° either in amplitude (Cobb's method) or apical torsion (Pedriolle's method) [14,15]. Three outcomes were distinguished based on changes in curve magnitude (C_M_) between baseline (t_0_) and the end of treatment (t_1_): curve correction (C_M _t_1_-t_0 _≤ -5° Cobb), curve stabilization (C_M _t_1_-t_0 _≥ -5 and ≤ 5° Cobb) and curve progression (C_M _t_1_-t_0 _> 5° Cobb).

### Determination of Quality of Life

Three questionnaires were utilized to determine QoL in the sample population: the SRS-22 [16], the BrQ, and the BSSQ (brace version). These questionnaires were chosen because of their user-friendliness, reliability, satisfactory internal consistency, reproducibility and responsiveness to changes in QoL in AIS patients treated with bracing [4-6,10-12]. Questionnaires were administered at least one year after treatment commencement and, in any cases, not during the weaning. Interviews were held in the waiting room, after medical evaluation, with a physician available for any elucidations. Briefly, the SRS-22 is comprised of 22 items exploring 5 domains pertinent to psychophysical wellbeing: function/activity level, pain, mental health, self image, treatment satisfaction. For each item, the score ranges from 0 (worst) to 5 (best) [10-13], with a summary score between 22 and 110. The BrQ comprises 34 items exploring 8 domains: overall health perception, physical function, emotional function, body aesthetic perception, vitality, school activity, pain, and social activity. For each item, the score ranges from 1 (best) to 5 (worst) and varies depending on the question ("always", "most of the times", "sometimes", "rarely", "never"). The summary score is calculated by multiplying each item by 20 and subsequently dividing the total score by 34. The BrQ summary score ranges from 100 (optimal QoL) to 20 (very poor QoL) [4]. The BSSQ in its "brace version" comprises 8 items, whose scores range from 0 to 3. The summary score is obtained by summing the score of the single items, and ranges from 0 (maximum stress) to 24 (minimum stress) [5,6].

### Preliminary validation of the Italian version of the BrQ and BSSQ questionnaires

Of the 3 questionnaires used, only the SRS-22 had already been validated in Italian [16]. For the other 2 instruments, a preliminary validation was conducted in a sample of 34 AIS patients. The sample was comprised of 30 girls and 4 boys, ranging in age between 11 and 16 years (mean: 15.4 years), with a curve severity at the time of questionnaire administration of 18.2° Cobb. Participants were prescribed full-time treatment with PASB (n = 14), Lyon brace (n = 17) or a combination of the two (n = 3). The validation process of the BrQ and BSSQ entailed two stages: first, a translation and a back translation were performed to develop the respective Italian versions; hence, the questionnaires were administered twice with a 5-7 days interval, and reliability and internal consistency determined. The SRS-22 was also administered as the reference questionnaire. All data were normally distributed, as indicated by the Kolmogorov-Smirnov's test. Mean summary scores at the BrQ and BSSQ were 77.3 and 12.8, respectively. The test-retest reliability was calculated by means of the Pearson's test and showed a high temporal stability for both the BrQ (r = 0.88; p < 0.001) and the BSSQ (r = 0.92; p < 0.001). Internal consistency was determined through the Cronbach's α coefficient. The overall Cronbach's α values for the BrQ and BSSQ were 0.86 and 0.91, respectively, indicating satisfactory internal consistency for both questionnaires.

### Statistics

Statistical analysis was performed using the SPSS software version 13.0. Normality of data was ascertained by the Kolmogorov-Smirnov's test. Differences among groups were assessed by t-test and ANOVA, as appropriate. Correlations between variables were explored by linear regression analysis. All data are presented as mean ± S.E.M. For data pertaining to curve severity, the median is also reported. For all tests statistical significance was set at p < 0.05.

## Results

The mean age of participants treated with PASB only was 15.7 ± 0.3 years, while the curve entity at the beginning of treatment and at the time of questionnaire administration was 28.0 ± 1.2° Cobb (median: 26°) and 13.3 ± 1.2° Cobb (median: 13°), respectively. Patients treated with the Lyon brace only were 15.4 ± 2.0 year old, with a curve severity at baseline and at the time of interview of 35.2 ± 1.6° Cobb (median: 30.0°) and 21.7 ± 1.6° Cobb (median: 19°), respectively. Hence, participants treated with the Lyon brace did not differ from those wearing the PASB in terms of age, but had higher curve severity both at baseline (p = 0.001) and at the time of questionnaire administration (p < 0.001) relative to PASB-treated patients.

Curve correction was accomplished in 95 patients (88%), whilst stabilization was obtained in 11 cases (10.2%). Two patients (1.8%) experienced a curve progression. Scores obtained by participants at the SRS-22, BrQ and BSSQ are shown in Table 1.

**Table 1:** Participants's scores at the BrQ, SRS-22 and BSSQ

	*Mean*	*S.E.M. (±)*
**Brace Questionnaire (BrQ)**		

Summary score	78.8	1.0
General health perception	3.5	0.1
Physical functioning	4.1	0.1
Emotional functioning	3.5	0.1
Self-esteem and aesthetics	3.7	0.1
Vitality	3.6	0.1
School activity	4.5	0.1
Bodily pain	4.1	0.1
Social functioning	4.1	0.1

**Scoliosis Research Society 22 (SRS-22)**		

Summary score	85.9	1.0
Satisfaction with management	8.6	0.1
Mental health	19.1	0.4
Self image/appearance	17.0	0.3
Pain	21.5	0.3
Function/Activity	19.7	0.3

**Bad Sobernheim Stress Questionnaire (BSSQ)**		

Summary score	12.6	0.5

**Figure 1:**
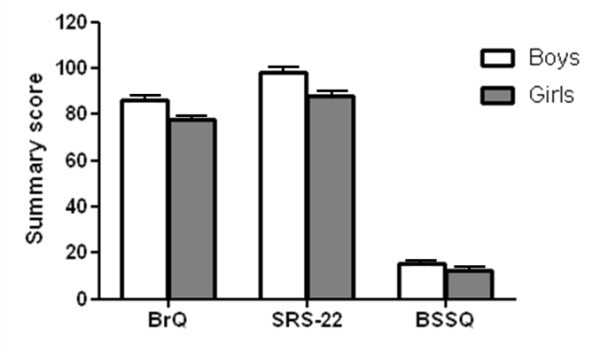
**Summary QoL scores according to participants' gender**.

**Figure 2:**
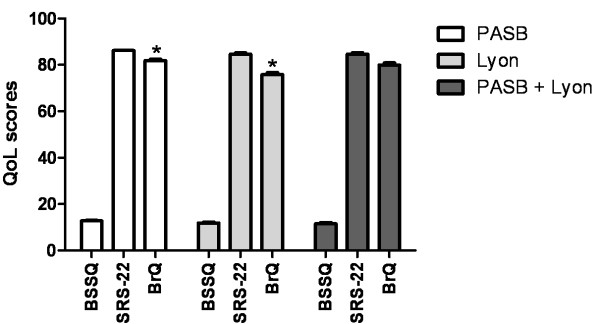
Summary QoL according to the type of bracing (* p < 0.05).

Overall, boys displayed better QoL than girls in all 3 questionnaires (SRS-22: p = 0.006; BrQ: p = 0.003; BSSQ: p = 0.04) (Figure 1). Regarding the type of bracing, patients treated with PASB only had higher QoL scores at the BrQ compared with those treated with Lyon alone (Figure 2). In contrast, no differences in QoL between the 2 braces emerged at the SRS-22 and BSSQ (Figure 2). In patients wearing PASB + Lyon brace, QoL did not significantly differ from that of either PASB or Lyon alone in any of the questionnaires.

**Figure 3:**
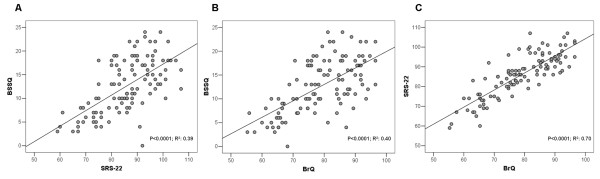
**Correlations among summary scores of the BSSQ, BrQ and SRS-22**.

The scores of the three questionnaires showed significant correlation with each other (Figure 3A-C). Furthermore, a significant correlation was detected between corresponding domains of the SRS-22 and BrQ (Table 2).

**Table 2:** Correlations between equivalent domains of the SRS-22 and BrQ questionnaires

Domains	P	**R**^**2**^
SRS-22 function/activity - BrQ social functioning	< 0.0001	0.52
SRS-22 function/activity - BrQ vitality	< 0.0001	0.48
SRS-22 self-image/appearance - BrQ self-esteem and aesthetics	< 0.0001	0.60
SRS-22 mental health - BrQ emotional functioning	< 0.0001	0.60
SRS-22 pain - BrQ bodily pain	< 0.0001	0.71

**Figure 4:**
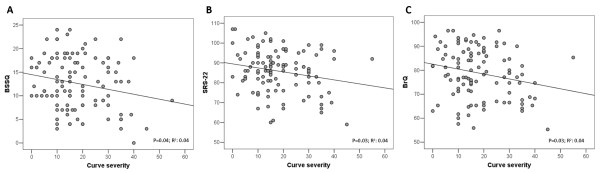
**Correlations between curve severity at the time of questionnaire administration and summary scores at the BSSQ (A), SRS-22 (B) and BrQ (C)**.

Summary QoL scores were correlated with the curve severity at baseline only in the BrQ (p = 0.02, R^2^: 0.02; data not shown). However, a weak yet significant correlation was determined for all 3 instruments at the time of questionnaire administration (Figure 4A-C). After dividing the sample population into tertiles of curve severity (0-30° Cobb; 31-50° Cobb, and > 50° Cobb) at the time of questionnaire administration, a reduced QoL was observed only in the BrQ (p < 0.01). Since the tertile with the most severe curves had a quite small numerosity, analyses were also performed after dividing the whole population in 2 severity groups: 0-30° Cobb (n = 93) and 31-70° Cobb (n = 15). Patients with milder curves displayed higher QoL scores at the BrQ (p < 0.01) and BSSQ (p < 0.05), but not at the SRS-22.

Our analyses did not show differences in QoL depending on the curve type (data not shown). However, patients with thoraco-lumbar curves were less satisfied with management than those with thoracic curves at the SRS-22 (p = 0.004; data not shown). In contrast, the BBSQ and BrQ showed an opposite pattern (p = 0.03 and p = 0.007, respectively; data not shown). Finally, no significant correlations were observed between QoL scores and the difference in curve severity between baseline and the time of questionnaire administration (data not shown). Similarly, no significant correlations were determined between improvements in Cobb angle and satisfaction with management (data not shown).

## Discussion

Results from the present study indicate that the SRS-22, BrQ and BSSQ are effective tools for the evaluation of QoL in AIS patients. Moreover, scores obtained in similar domains of the SRS-22 and BrQ are highly concordant with one another. However, the BrQ appeared to be superior in capturing changes in QoL according to the type of bracing, curve severity, curve type and gender compared to the other questionnaires. This finding might be related to the higher number of questions of the BrQ, which may enable it to explore more domains than the other questionnaires. Therefore, although the administration of BrQ requires more time and concentration compared with the SRS-22 and the BSSQ [4], our study supports its use, given the relevance of QoL to the treatment acceptance and compliance. Importantly, our results highlight the effectiveness of conservative treatment for AIS, without severe QoL deterioration. This is especially true for the PASB [17], likely as a result of its innovative design and reduced dimensions as opposed to the Lyon brace. Finally, in our population, boys displayed a better QoL than girls, probably because of the higher concern about physical appearance experienced by young girls.

The effectiveness of the brace treatment in AIS is widely acknowledged [17-19] and is further supported by the present study. However, conservative treatment may significantly impact on patients' psychological wellbeing and negatively affect their QoL. Previous studies have produced mixed results with regards to the effect of bracing on QoL. Indeed, some authors reported brace treatment to be associated with high levels of stress and poor QoL [20-23]. For instance, Freidel and co-workers [24] found a high prevalence of depressed mood and reduced QoL in brace-treated AIS patients. Furthermore, bracing has been associated with negative body perception, reduced self-esteem, increased levels of stress and higher susceptibility to develop phobias and anxiety compared to surgical treatment [25]. Interestingly, Kotwicki and colleagues [6] noticed that levels of stress in AIS patients were higher when they were asked about their braces as opposed to their deformities. This finding highlights the difficulties AIS patients experience when subjected to conservative treatment, indicating that QoL should be carefully monitored over the course of treatment.

Other investigations did not report deteriorations in QoL in brace-treated AIS patients [7,26,27]. For instance, Olafsson et al. [28] found that bracing did not negatively affect self-image perceived by adolescent patients. It was also reported that bracing may have a psychological impact at the beginning of treatment, evidenced by reduced self-esteem [21]. However, no different rates of psychopathologies were observed between brace-treated patients and age-matched healthy controls [21]. Finally, Noonan and co-workers [27] reported small differences in psychological wellbeing between scoliotic adolescents treated with bracing and healthy controls. Moreover, these differences tended to vanish in the adulthood [27].

With regards to QoL evaluation in AIS patients, several instruments have been proposed over the last years. The SF-36 has been the first tool used for this purpose [29]. However, the SF-36 is not designed for scoliosis and may therefore be inadequate to capture the complex interaction among QoL, scoliosis and scoliotic treatment. The SRS-22 was proposed as alternative instrument to overcome the limitations intrinsic to the SF-36 [10,12,30]. This tool explores several domains and has proven valid, easy-to-use, comprehensible, responsive to changes and with good concurrent validity [10,13,29]. The SRS-22 score has been reported to be inversely correlated with the curve severity [13]. However, the SRS-22 was not correlated with the curve type or the presence of single or multiple curves [13]. In contrast, in our study, the SRS-22 was able to discriminate changes in QoL according to the gender, but not as a function of type of bracing, curve type and curve severity. However, the SRS-22 was correlated with the curve severity at the time of questionnaire administration.

Regarding the BrQ, it was previously shown to represent a reliable and valid tool to evaluate QoL in AIS patients, as indicated by its responsiveness to changes in QoL and its correlation with the curve severity [4]. Results from our studies support the validity of the BrQ, by showing that the questionnaire is able to differentiate the QoL according to gender and curve severity. However, scores at the BrQ were not different depending on the type of bracing and curve.

Similar to the BrQ, the BSSQ represents a useful and easy-to-use tool to explore QoL in AIS patients. Previous studies reported the BSSQ score to be correlated with the severity of clinical and radiographic deformities. Moreover, results of the BSSQ are influenced by the type of treatment, indicating that the questionnaire is highly sensitive to objective AIS parameters [4,5]. Studies employing the BSSQ have shown moderate levels of stress in brace-treated AIS patients, with summary scores ranging from 9 to 13.9 [6,31], which are in keeping with our results.

The type of bracing utilized has a great impact on patients' QoL, depending on the design, dimensions, degree of physical restrain and visibility. For instance, the Milwaukee has been associated with greater deteriorations in QoL compared with the Boston or thoracolumbosacral orthosis (TLSO) such as the Charleston bending brace [3]. Moreover, the use of light braces such as the Cheneau light is associated with reduced levels of stress compared with heavier bracing (e.g., Cheneau brace, Boston brace, Cheneau Boston-Wiesbaden brace, Wilmington brace, SpineCor brace) [31]. In our study, patients treated with the PASB displayed a better QoL than those treated with the Lyon brace, possibly due to the fact that the first is less visible and better tolerated than the Lyon brace. In addition, in patients treated with the Lyon brace, the curve severity both at baseline and at the time of interview was higher than in those treated with the PASB, which might have partly contributed to our finding.

Concerning the impact of gender on QoL, our results indicate that girls experience a greater deterioration in QoL compared with boys. This finding is in agreement with previous studies, also reporting a better QoL in brace-treated AIS males. For instance, Freidel et al. [32] found that the prevalence of depressive symptoms and negative feelings toward life was higher in female patients. Furthermore, Korovessis at al. [33] reported a 9% rate of brace treatment discontinuation among girls because of psychological distress. Interestingly, Olafsson et al. [28] showed that gender-related differences in QoL amplify with increasing age, with the maximum separation between males and females at about 20 years. This phenomenon has been attributed to the growing concern about aesthetics experienced by adolescent and young-adult females, in contrast to the improvement in self-perception experienced over time by males.

In our case series, we did not detect differences in QoL depending on the curve type. This is keeping with the study by Asher et al. [13], where no correlation was determined between the type of curve and QoL. However, other investigators showed that patients with thoraco-lumbar curves had worse QoL scores than those with thoracic curves [34], probably as a consequence of the milder aesthetic impact of thoraco-lumbar scoliosis. Similarly, patients with thoraco-lumbar curves were less satisfied with management than those with thoracic curves [34]. This finding is supported in our study by the SRS-22. In contrast, the BBSQ and BrQ showed an opposite pattern. This discrepancy underlines the complexity of QoL evaluation in AIS patients and the need for carefully monitoring this parameter over the course of treatment. Finally, Bunge and coworkers [34] reported a weak, but significant correlation between improvements in Cobb angle and satisfaction with management, which was not observed in our study.

## Conclusions

In summary, findings from the present study suggest that conservative treatment does not severely impact on QoL of AIS patients. Nevertheless, close QoL monitoring should be routinely implemented during brace treatment, taking into account gender, type of bracing, curve type and severity, in order to provide psychological support if needed. This approach may increase the compliance to treatment, which is instrumental for a successful outcome. Finally, our data indicate that the 3 questionnaires tested and especially the BrQ are effective tools for monitoring QoL in brace-treated patients.

## Abbreviations

AIS: Adolescent Idiopathic Scoliosis; BrQ: Brace Questionnaire; BSSQ: Bad Sobernheim Stress Questionnaire; HRQoL: Health Related Quality Of Life; PASB: Progressive Action Short Brace; QoL: Quality of Life; SRS-22: Scoliosis Research Society 22; TLSO: Thoraco-Lumbar-Sacral Orthosis.

## Competing interests

The authors declare that they have no competing interests.

## Authors' contributions

All authors contributed equally to this work; all authors read and approved the final manuscript.
